# The gluten-free diet challenge in adults with coeliac disease: the Hellenic survey

**DOI:** 10.1016/j.pecinn.2022.100037

**Published:** 2022-03-31

**Authors:** Spyridaki Aspasia, Kotsoni Emmanuela-Kalliopi, Thalassinos Nikolaos, Sfakianaki Eirini, Sfendourakis Ioannis, Markaki Anastasia

**Affiliations:** Department of Nutrition and Dietetics Sciences, Hellenic Mediterranean University (HMU), Trypitos area, 723 00 Sitia, Greece

**Keywords:** Coeliac disease, gluten-free diet, dietary adherence, information sources

## Abstract

**Objective:**

The only available treatment for coeliac disease (CD) is a lifelong strict gluten-free diet (GFD), which can be extremely challenging. Τhe aim of the present study was to gain an insight into patients' perceptions regarding the GFD, in relation to difficulties experienced, disease-specific symptoms, adherence level, and information sources used.

**Methods:**

Two hundred ninety CD patients (247 women and 43 men) aged 18–74 years, completed a self-administered questionnaire.

**Results:**

Self-rated dietary adherence was high in 65.5%, moderate in 27.5% and poor in 6.9% of the patients*.* The main difficulties encountered were the high cost and limited availability of GF foods and meals in markets and restaurants. The main source of information concerning CD and GFD was the internet, and the most useful source was the Coeliac Society. Dietary adherence correlated positively with Coeliac Society membership and awareness of the monthly CD allowance.

**Conclusion:**

Α substantial proportion of patients did not adhere to a strict GFD. Patients were not adequately followed-up.

**Innovation:**

A self-administered questionnaire was used to explore the practical challenges of a GFD in an understudied population, Greek adult coeliac patients. The results highlighted the need for dietitians with expertise on CD.

## 1 Introduction

Coeliac disease (CD) is an immune-mediated disease characterized by damage to small intestinal mucosa by exposure to dietary gluten in genetically susceptible people [1]. It has a broad spectrum of clinical presentations, ranging from classical gastrointestinal or extra-intestinal symptoms to asymptomatic forms. Over the past two decades, CD has emerged as a major public health problem, affecting approximately 1% of the European population [2].

To date, the only available treatment for CD is strict, lifelong adherence to a gluten-free diet (GFD) [3]. According to Catassi et al. (2007), an intake of as little as 50 mg gluten/day (equivalent to a few breadcrumbs) is sufficient to cause significant mucosal deterioration in CD patients [4]. Insufficient adherence to a GFD in CD patients is associated with an increased rate of anemia, osteoporosis, infertility, secondary autoimmunity, malignancies, and mortality [5].

However, adhering to a GFD can be extremely challenging because gluten is an ingredient commonly found in most diets [6]. Several studies have demonstrated that GFD adherence among CD patients is insufficient [7,8]. Factors which greatly influence GFD adherence include understanding of the GFD and food labeling, as well as cost and availability of GF foods [9,10]. Therefore, it is crucial that CD patients are offered accurate and comprehensive information about the GFD and followed up with a healthcare professional on a regular basis to assure long-term dietary adherence [5].

Given the paucity of information on GFD management in Greek adult CD patients, the aim of the present study was to gain an insight into patients' perceptions regarding the GFD, in relation to level of adherence, difficulties experienced, disease-specific symptoms, and sources of information used, in order to identify challenges of managing a GFD in this patient population.

## 2 Methods

### 2.1 Survey description

A 33-item self-administered questionnaire was developed in Greek language*.* The questionnaire consisted of four sections which involved demographics (9 questions)*,* disease-related information (4 questions), 9 questions relating to *GFD* (e.g. adherence, persisting symptoms, perceived difficulties), as well as a section on information sources. The latter section had one question on patients' main source of information on CD and GFD, and a list of 10 commonly used sources of information for CD patients, according to previous studies [[11], [12], [13]]*.* Participants were asked to rate each source as “not useful”, “very useful” or “essential”.

The questionnaire was distributed online to the Coeliac Society and Facebook support groups. The questionnaire was filled out through an online survey platform (Google Forms). An information sheet was set as the first page of the online survey, which included a summary of the project*,* the aim of the survey and the target population. It confirmed that participation in the survey was voluntary and anonymous. Individuals who checked the informed consent box were directed to the survey's questions. The survey was open for completion for approximately 1 month (from 14 November 2019 until 10 December 2019). The inclusion criteria were a) individuals with CD, b) adults (≥ 18 years old) and c) Greek residents.

### 2.2 Statistical analyses

For statistical analysis the Statistical Package for the Social Sciences (SPSS), version 23.0 was used. Nominal and ordinal data were summarized as absolute frequencies and percentages. Continuous variables were summarized as mean and standard deviation. Categorical variables were compared with the chi square test. *P*-values below 0.05 were considered statistically significant. Quantitative variables were compared with the Student *t-*test in case of a normal distribution or otherwise with the Mann-Whitney *U* and Kruskal–Wallis nonparametric tests. Also, Pearson and Spearman tests were used to examine correlations between continuous variables with normal and abnormal distribution.

## 3 Results

A total of 307 individuals responded to the online survey, 294 (95.8%) met the inclusion criteria and 290 (94.5%) fully completed the questionnaire. Thus, the study included 290 subjects*,* 247 females and 43 males. Mean age of the respondents was 38.86 ± 11.25 years. Mean age of diagnosis was 28.50 ± 13.12 years. The sociodemographic and clinical characteristics of the respondents are summarized in Table 1, Table 2, respectively. Most respondents (81.7%) were highly educated (with at least a bachelor's degree)*,* were members of the Coeliac Society (63.8%) and were aware of the monthly allowance for CD patients (89.0%). The majority of respondents had no family history of CD (77.9%), were informed of their disease by a doctor (89.3%) and were diagnosed via small bowel biopsy (81.7%). Five point two% of the respondents were self-diagnosed. Most common symptoms prior to diagnosis were abdominal pain/bloating (57.9%), diarrhea/constipation (57.2%), fatigue (45.2%), and vomiting/weight loss (32.4%). Of note, 13.1% of the respondents had no symptoms prior to diagnosis.

**Table 1:** Sociodemographic characteristics of respondents.

Variable	**n (%)**
Gender	
Female	247 (85.2)
Male	43 (14.8)
Education level	
Secondary Education	47 (16.2)
Bachelor	169 (58.3)
Master- Ph.D.	68 (23.4)
Other	6 (2.1)
Civil Marital status	
Single	96 (33.1)
Married	173 (59.7)
Other	21 (7.2)
Monthly income	
>1000 euro	76 (26.2)
500–1000 euro	125 (43.1)
<500 euro	89 (30.7)
Place of residence	
Urban area	194 (66.9)
Rural area	96 (33.1)
Members of the Hellenic Coeliac Society	
Yes	185 (63.8)
No	105 (36.2)
Were aware of the monthly allowance for CD patients	
Yes	258 (89.0)
No	32 (11.0)

CD: Coeliac disease.

**Table 2:** Clinical characteristics of respondents.

	**n (%)**
Person who made diagnosis	
by a doctor	259 (89.3)
self-diagnosis	15 (5.2)
dietitian-nutritionist	6 (2.1)
internet	3 (1.0)
other	7 (2.4)
Method of CD diagnosis	
small bowel biopsy	237 (81.7)
blood test for CD	38(13.1)
other	15 (5.2)
Symptoms prior to diagnosis	
Abdominal pain-Bloating	168 (57.9)
Diarrhea-Constipation	166 (57.2)
Fatigue	131 (45.2)
Vomiting-Weight loss	94 (32,4)
Skin rash	56 (19.3)
Other	95 (32.8)
Family history of CD	
Yes	64 (22.1)
No	226 (77.9)

CD: Coeliac disease.

GFD-related characteristics are presented in Table 3. The GFD was recommended by a doctor in 82.8% and a dietitian in 10.3% of the respondents (less frequent replies, “other”, included self-recommended, friend, and other person with CD). Thirty-two point eight% of the respondents remained symptomatic despite being on a GFD, of which 29% reported partial symptom resolution.

**Table 3:** GFD-related characteristics of respondents.

	**n (%)**
Person who recommended a GFD	
Doctor	240 (82.8)
Dietitian	30 (10.3)
Other	20 (6.9)
Read food labels	
Yes	277 (95.5)
No	13 (4.5)
Consider reading food labels helpful	
Yes	265 (91.4)
No	25 (8.6)
*Have* difficulty in finding GF products in the market.	
Yes	170 (58.6)
No	120 (41.4)
Have difficulty in finding gluten-free meals at restaurants	
Yes	272 (93.8)
No	18 (6.2)
Self-rated degree of adherence to GFD	
High	190 (65.5)
Moderate	80 (27.5)
Poor	20 (6.9)
Have symptoms despite adherence to GFD	
Yes	11 (3.8)
Yes, but less	84 (29.0)
No	195 (67.2)
Find it difficult to follow a GFD	
Yes	224 (77.2)
No	66 (22.8)
Find the GFD difficult to follow because:	
Of limited availability	182 (81.3)
Of the cost	148 (66.1)
It is time-consuming	69 (30.8)

GFD: Gluten-free diet; GF: Gluten-free.

The participants were asked to rate themselves as highly/moderately/or poorly compliant (Question: How compliant are you in following a GFD?). Accordingly, the obtained level of GFD adherence was high in 65.5%, moderate in 27.5% and poor in 6.9% of the respondents. The vast majority read food labels (95.5%) and considered them useful (91.4%). Still, most respondents (77.2%, *n* = 224) found the GFD difficult to follow, namely due to the limited availability (81.3%, *n* = 182) and cost of gluten free food (66.1%, *n* = 148). Fifty-eight point six% of the respondents reported that they have difficulty in finding GF products in the market and 93.8% that they have difficulty in finding gluten-free meals at restaurants.

The main source of information used to acquire information on CD and GFD was the internet for 54.5% (*n* = 158) of the respondents. Doctors were cited as the most utilized source of information by 25.5% (*n* = 74) and dietitians-nutritionists by 7.9% (*n* = 23) of the respondents. Furthermore, scientific meetings-conferences were the primary source of information for 9% (*n* = 26) of the respondents, and food companies for 3.1% (*n* = 9).

**Fig. 1:**
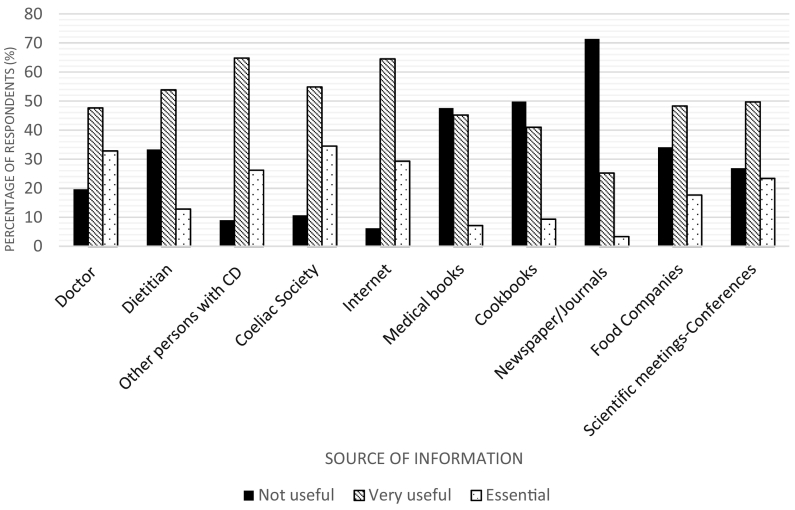
Perceived usefulness of information about coeliac disease and gluten-free diet according to source. CD: Coeliac disease.

The questionnaire also included a list of ten commonly used information sources on CD and GFD [[11], [12], [13]]*,* and participants rated their usefulness (Fig. 1). The highest ratings (rated as essential by the respondents) were the following: Coeliac Society *(n* *=* 100, 34.5*%),* doctor *(n* *=* 95, 32.8*%),* internet *(n* *=* 85, 29.3*%),* other persons with CD *(n* *=* 76, 26.2*%),* scientific meetings-conferences *(n* *=* 68, 23.4*%),* food companies *(n* *=* 51, 17.6*%),* dietitian *(n* *=* 37, 12.8*%),* medical books (*n* = 21, 7.2%), cookbooks (*n* = 27, 9.3%) and newspaper/journals (n = 10, 3.4%).

Furthermore, the survey revealed several statistically significant correlations. Having difficulty in following a GFD correlated positively with the difficulty in finding GF products in the market (*p* < 0.001) and GF meals at restaurants (p < 0.001), and negatively with monthly income (*p* = 0.049) and being a member of the Coeliac Society (*p* = 0.007). Coeliac Society membership correlated positively with GFD adherence (*p* = 0.001) and awareness of the monthly CD allowance for GF products (*p* = 0.006). GFD adherence was also associated with awareness of the monthly CD allowance (p < 0.001). We did not find an association between GFD adherence and age of the respondents or age at diagnosis.

## 4 Discussion and conclusion

### 4.1 Discussion

The present web-based survey, which included 290 Greek adults with CD, demonstrated that at least one-third of the respondents did not follow a strict GFD*.* Estimates of the rate of dietary adherence in the literature vary from 42% to 91% depending on the definition of ‘strict diet’ and the assessment method [7]. Although the estimated rate of GFD adherence in our study was within the aforementioned range, it most likely represents a significant overate of true dietary adherence, since inadvertent gluten intake was not taken into account [14]. The consequences of maintaining gluten in the diet include development of anemia, osteoporosis, secondary autoimmunity, malignancy, and increased risk of mortality [5].

Good knowledge of the disease and the GFD and understanding food labels are considered key factors to achieve good dietary adherence [9]. Contrary to previous studies, which report difficulties in reading food labels among CD patients [10,11], the vast majority of our respondents read food labels and considered them useful. This could be attributed to the fact that most respondents were highly educated and Coeliac Society members, which are generally more informed and motivated patients [12]. Still, most of the respondents found the GFD difficult to follow. The main difficulties encountered by the respondents were the high cost and limited availability of GF food, which is in line with previous studies [6,15]. Furthermore, a major difficulty encountered by almost all respondents was finding GF meals at restaurants. Based on previous findings, exposure to gluten while dining out is a leading source of anxiety for CD patients, since they should be certain that chefs and restaurant staff are well–informed about the need for the meal to be gluten–free and free of cross–contamination [16].

Recent studies have demonstrated that the GFD imposes a significant economic burden on CD patients [17,18]. Μany healthcare systems around the world have adopted various approaches to compensate for the additional costs of purchasing GF foods in order tο encourage GFD adherence and reduce food inaccessibility among CD patients [[19], [20], [21]]. For example, in Greece CD patients are provided with a monthly allowance [22], which was linked to higher self-rated GFD adherence among the patients of the present study. Moreover, having difficulty in following a GFD correlated negatively with the respondents' monthly income.

Successful management of CD requires significant patient education, motivation and follow-up, as underlined by the European Society for the Study of Coeliac Disease guideline [23]. Ideally, information on GFD should be given in collaboration with a dietitian, right after being diagnosed with CD. Inadequate advice at this stage may lead some patients to seek answers from less reliable sources, as there are currently many information sources available for patients in various forms [13]. The present survey revealed that the internet was the main source of information on CD and GFD for the majority of respondents. The internet is increasingly being used for health-related purposes; a trend termed the e-patient revolution [[24], [25], [26]]. Although the internet is an easily accessible and inexpensive source, the information on many websites addressing CD is not always accurate and is not considered sufficiently trustworthy and reliable for CD patients [27,28]. Furthermore, patient support is not a one-way communication but should be a dialogue between a healthcare professional and the patient [12]. One way to promote correct information is for healthcare professional to direct the patient to more reputable sites in order to ensure that the patient has the greatest chance of accessing accurate information.

According to the literature many people refer to online sources to self-diagnose and use these sources to determine a treatment plan [28,29]. In fact, 5.2% of the respondents of the present study belonged to the “self-diagnosed” category. It should be emphasized that a GFD is not an appropriate choice without a medical diagnosis [30,31]. Despite, the popular belief that gluten restriction represents a healthy lifestyle for the general population, commercial gluten-free products often have compromised nutritional quality, compared to their gluten-containing equivalents and a self-adapted GFD without the support of a dietitian/nutritionist may lead to nutritional deficiencies and metabolic disorders [[31], [32], [33]].

In our study, doctors were the second most frequently used source of information and only a minority relied on dietitians for disease-related nutrition information. A recent survey conducted by Miaja et al., demonstrated that the most frequently used sources of information were the internet and CD associations, in addition to books and other CD patients, while physicians and dietitians were the least frequent choices [34]. These findings indicate the lack of appropriate support from healthcare professionals.

Although long-term follow-up is essential to assure treatment adherence and positive health outcomes, previous studies have shown that CD patients are not consistently followed up after diagnosis [23,35]. A study in the USA, which included 413 CD patients, found that despite referral to see a dietitian, only 79% of those diagnosed with coeliac disease had seen a dietitian, and of those who had seen a dietitian, 39% had only one consultation [36]. In another survey on CD patients, only 13% of respondents reported that they had received information from a dietitian and of those only 21% found it useful [37]. A survey conducted by Green, and colleagues found that while many patients are referred to and advised by a dietitian, the most useful information that they receive is provided by coeliac support groups [38].

When the participants of the present study were asked to rate the usefulness of the information on CD and GFD obtained by various common sources, the coeliac society, doctors, the internet and other persons with CD received the highest ratings. Previous surveys have ascertained the important role played by coeliac societies in providing CD patients with practical advice, information, and support [39,40]. Membership of CD support groups has been linked to higher dietary adherence. Our study demonstrated that CD membership correlated positively with self-rated GFD adherence and awareness of the CD allowance and negatively with having difficulty in following a GFD, which could explain why this organization received the highest rating.

Concerning the low ratings of the usefulness of information provided by dietitians, our findings are similar to those reported elsewhere [11,13]. According to a Canadian study on CD patients, perceived usefulness of the information obtained on CD ranged from 90.4% (Coeliac Support Association), 66.9%, (another person with CD), 62.0% (cookbooks), 53.2% (internet), 52.1% (dietitian), 50.7% (medical books/journals), 47.0% (alternative medical professional), 42.9% (gastroenterologist), 28.4% (newspaper/magazine) to 25.3% (family doctor). In another study, patient advocacy groups, other persons with CD, cookbooks, the internet, and gastroenterologists were identified as the top five most useful sources of information about the GFD [13].

These results underline the need to improve education of dietitians about CD and GFD. It should be mentioned, however, that dietary consultations in Greece are not reimbursed and most public hospitals have a limited number of dietitians. Therefore, the low ratings may also reflect that few respondents received dietary information from the specific source. In any case, diagnosed patients should be referred to an expert registered dietitian for accurate GFD education and follow-up in order to reinforce adherence and to reduce the risk of complications associated with an inadequately balanced GFD. The incidence of CD is rising globally, and the healthcare community should be adequately prepared [41]. As dietary intervention is the only available treatment option, there is an important role for dietitians to play [42].

This study has certain limitations that should be considered. Firstly, selection bias may exist as participants were recruited mainly through coeliac support groups. Also, as an online survey, it required digital literacy skills and internet access. Furthermore, the study relied on self-reported information for all data. Clearly, further research is necessary to estimate the rate of GFD-adherence among Greek CD adults more accurately, based on objective methods such as the recently developed test that determines gluten consumption by assessing gluten-derived peptides in human samples [43]. Nevertheless, this is the first study, to our knowledge, to address GFD adherence and perceived difficulties among Greek adults with CD and it demonstrates the lack of adequate patient follow-up by dietitians. Since the GFD is the cornerstone treatment for CD, diagnosed patients should be referred to a dietitian for dietary counseling and follow-up to reinforce adherence to a balanced GFD. Consequently, registered dietitians with expertise on CD are required. Furthermore, government policies are needed to increase the availability and reduce the cost of GF foods, to promote awareness of GF food in catering staff and to enhance patients' access to dietitians with expertise on CD.

### 4.2 Innovation

A 33-item self-administered questionnaire was designed in an attempt to capture the collective voice of an understudied population, Greek adult coeliac patients. The survey focused on patients' perceptions regarding the GFD in relation to difficulties experienced, disease-specific symptoms, adherence level, and information sources used. The study provided insight into the practical challenges faced by patients in adhering to a lifelong strict GFD and highlighted the need for dietitians with expertise on CD.

### 4.3 Conclusion

The study revealed that a substantial proportion of patients did not adhere to a strict GFD. Financial issues and availability of GF foods were found to be major barriers to GFD adherence. Patient follow-up by dietitians was inadequate and the majority of patients relied on the internet to gain disease-related nutrition information.

## Authors' contributions

All authors have contributed to the design of the study, data collection and analysis and writing the manuscript.

## Funding

The authors report no funding.

## Declaration of Competing Interest

The authors declare that they have no known competing financial interests or personal relationships that could have appeared to influence the work reported in this paper.
